# Cantharidin suppressed breast cancer MDA-MB-231 cell growth and migration by inhibiting MAPK signaling pathway

**DOI:** 10.1590/1414-431X20175920

**Published:** 2017-07-03

**Authors:** X.-D. Gu, L.-L Xu, H. Zhao, J.-Z Gu, X.-H Xie

**Affiliations:** 1 Department of Breast Surgery, First Affiliated Hospital of Zhejiang Chinese Medical University, Hangzhou, Zhejiang, China; 2 First Clinical Medical School, Zhejiang Chinese Medical University, Hangzhou, Zhejiang, China; 3 Oncology Department, First Affiliated Hospital of Zhejiang Chinese Medical University, Hangzhou, Zhejiang, China

**Keywords:** Cantharidin, Breast cancer, ERK/MAPK pathway, Growth, Migration, Invasion

## Abstract

As an active constituent of the beetle *Mylabris* used in traditional Chinese medicine, cantharidin is a potent and selective inhibitor of protein phosphatase 2A (PP2A) that plays a crucial role in cell cycle progression, apoptosis, and cell fate. The role and possible mechanisms exerted by cantharidin in cell growth and metastasis of breast cancer were investigated in this study. Cantharidin was found to inhibit cell viability and clonogenic potential in a time- and dose-dependent manner. Cell cycle analysis revealed that cell percentage in G2/M phase decreased, whereas cells in S and G1 phases progressively accumulated with the increasing doses of cantharidin treatment. In a xenograft model of breast cancer, cantharidin inhibited tumor growth in a dose-dependent manner. Moreover, high doses of cantharidin treatment inhibited cell migration in wound and healing assay and downregulated protein levels of major matrix metalloproteinases (MMP)-2 and MMP-9. MDA-MB-231 cell migration and invasion were dose-dependently inhibited by cantharidin treatment. Interestingly, the members of the mitogen-activated protein kinase (MAPK) signaling family were less phosphorylated as the cantharidin dose increased. Cantharidin was hypothesized to exert its anticancer effect through the MAPK signaling pathway. The data of this study also highlighted the possibility of using PP2A as a therapeutic target for breast cancer treatment.

## Introduction

Breast cancer remains a health threat for females worldwide. According to a recent cancer statistic in the United States, breast cancer is the most frequently diagnosed cancer and the leading cause of cancer-related deaths among females; it accounts for 232,340 of the total cancer cases and 39,620 of cancer deaths, making it the most common non-skin cancer in females (1). Multiple factors, including histone modification (2,3), hormone disorder (4), and transcription factors (5), are considered to influence the development and progression of breast cancer. Hence, current knowledge considers breast cancer as a multistep disease that involves the coordinated interaction of multiple genes and the accumulation of multiple molecular and morphologic changes within a cell (6). Treatment modalities have progressed over the last decades; however, the prognosis of breast cancer patients is still poor. Therefore, a novel therapeutic strategy for treating breast cancer is urgently needed.

*Mylabris* is a Chinese blister beetle. Its dried body has been used as a traditional Chinese medicine to treat tumors for over 2,000 years and is still used as a folk medicine (7). The active constituent of *Mylabris* is cantharidin (8). Recently, cantharidin has received sudden research expansion and has become a major focus in anticancer research. The latest studies have found that cantharidin and its derivatives have potent anticancer activities in various types of cancer, such as hepatoma (9), lung cancer (10), gastric cancer (11), pancreatic cancer (8,12,13), bladder cancer (14,15), epidermoid carcinoma (16), and tongue squamous cell carcinoma (17). Clinical trials have inspiringly shown that cantharidin and its analogs have therapeutic effects against primary hepatoma and advanced stage cases (7,18). The broad-spectrum anticancer activity implies that cantharidin might play an important role in human cancers and might be a promising anticancer agent.

Mechanistically, cantharidin is a potent and selective inhibitor of protein phosphatase 2A (PP2A) – a multimeric serine/threonine phosphatase that can dephosphorylate multiple kinases (8), and play crucial roles in the control of cell cycle progression, apoptosis, and cell fate (19). Cantharidin inhibits PP2A and thereby phosphorylates multiple kinases and activates these kinase-mediated pathways. Consequently, cantharidin has exerted wide roles during tumor growth suppression, including the promotion of cell apoptosis and cell cycle arrest (10,11,14,17), and the induction of DNA damage (10). Cantharidin also inhibits cell migration and invasion through accelerated degradation of matrix metalloproteinase (MMP) (12,20).

However, knowledge about the role of cantharidin in anticancer activity is limited. Moreover, the signaling pathway that accounts for the anticancer property of cantharidin has rarely been studied. One pioneer study indicated that cantharidin-induced cell apoptosis is associated with the activation of mitogen-activated protein kinase (MAPK) pathways (21). In view of the nature of dephosphorylating kinases, this study hypothesized that the functional role of cantharidin might be closely related to kinase pathways. Therefore, this study aimed to 1) investigate the possible anticancer effect of cantharidin on breast cancer cells and 2) explore the underlying mechanisms associated with this activity, specifically focusing on MAPK pathways.

## Material and Methods

### Reagents and cell cultures

Cantharidin was purchased from Enzo Life Sciences International (USA). Primary antibodies against reduced glyceraldehyde-phosphate dehydrogenase (GAPDH), β-actin, matrix metalloproteinase-2 (MMP-2) and MMP-9 were purchased from Abcam (China). Antibodies against MEK, ERK, JNK and p38 MAPK were obtained from Cellular Signaling Co. (USA). Human breast cancer cell line MDA-MB-231 was purchased from the American Type Culture Collection (USA) and maintained in Dulbecco's Modified Eagle medium (DMEM) (Gibco, USA) containing 10% fetal bovine serum (FBS; Hyclone, USA), 100 IU/mL penicillin, and 100 mg/mL streptomycin. Cells were incubated in a humidified atmosphere at 37°C with 5% CO_2_. Cells were passaged every 2 days to obtain an exponential growth.

### Western blot analysis

Total cellular proteins were extracted using a lysis buffer. After quantification using a BCA kit (Beyotime, China), an equal amount of 50 ng proteins were loaded to a 12% SDS-polyacrylamide gel electrophoresis (SDS-PAGE) and then transferred to nitrocellulose membranes for 2 h at 300 mA using a transfer system. Membranes were thereafter blocked with 5% skim milk in tris buffered solution containing 1% tween 20 (TBST). Membranes were then incubated with corresponding primary antibodies. The blots were developed with secondary antibodies at room temperature for 1 h and enhanced with chemiluminescence (ECL) detection system.

### Cell viability assay

Cell viability upon cantharidin treatment was evaluated using the 3[4,5dimethylthiazol2yl]-2,5diphenyl tetrazolium bromide (MTT, Sigma, USA) assay. MDA-MB-231 cells were seeded onto 96-well plates at an initial 5×10^4^ cells/well. Cells were then co-incubated with cantharidin of different doses (0, 2.5, 5, 10, and 20 μM) for 48 h or incubated with 5 μM of cantharidin for 1, 2, and 3 days. For each monitored time, 10 µL of MTT reagent was added to each well at a final concentration of 0.5 mg/mL. The cells were then incubated at 37°C for another 4 h. Cell culture medium was then removed and replaced by 200 µL of dimethyl sulfoxide (DMSO) per well. The absorbance of each group of cells was determined at 490 nm using a microplate ELISA reader (Bio-Rad Laboratories, USA).

### Colony formation assay

Plate colony formation assay was conducted to evaluate the effect of cantharidin on anchorage-dependent cell growth. In brief, MDA-MB-231 cells were seeded at a density of 100 cells/well in 6-well plates, and were cultured in medium containing cantharidin with the indicated doses. After 10 days of free growth, the cells were washed with phosphate buffered solution (PBS) twice and stained with crystal violet. Any clone with over 50 cells was considered the real one. The number and size of visible colonies was manually counted for each group.

### Cell cycle analysis

Prior to analysis, serum was deprived for 24 h to synchronize cell cycle. Serum was then added back into the culture medium containing various doses of cantharidin (0, 2.5, 5, and 10 μM). After the treatments with cantharidin for 24 hours, MDA-MB-231 cells were fixed with 80% cold ethanol, and incubated with 0.5% Triton X-100 solution containing 1 mg/mL RNase A at 37°C for 30 min. Afterwards, propidium iodide (PI, Sigma) was added into each well at a final concentration of 50 μg/mL and the cells were further incubated for 30 min in the dark. Cell cycle progression was analyzed by a FACS (Becton Dickinson, USA). Data were processed using WinMDI29 software (Becton Dickinson).

### Wound healing assay

Wound-healing assays were explored by creating identical wound areas for anchorage-dependent MDA-MB-231 cells by 10-µL sterile pipette tips. Cells were seeded onto 6-well plates and co-incubated with different concentrations of Cantharidin (0, 2.5, 5, and 10 μM) for 24 h. Afterwards, cells were washed with PBS and scraped a vertical cross of constant width in the center of each well. MDA-MB-231 cells were then washed with PBS three times to remove detached cells and the culture medium was replaced by fresh serum-free medium immediately. After 24 h growth, cells in the scraped wound were observed and photographed under a Nikon microscope at a magnification of 200× for each group.

### Transwell migration and invasion assays

The transwell chambers with a polycarbonate filter (8-μm pore size) were purchased from Corning Co. (USA). For the transwell migration assays, MDA-MB-231 cells with the indicated doses of cantharidin were trypsinized and washed three times with FBS-free DMEM medium. Cells were then re-suspended in FBS-free DMEM at a density of 2×10^6^ cells/mL and seeded onto the upper chambers (100 μL). For the lower chambers, 600 mL of DMEM medium containing 10% FBS was added. Cells were then allowed to migrate for 12 h and membranes were thereafter stained with crystal violet. Cells that had transmigrated to the under surface of the filter were manually counted using a light microscope in five randomly selected fields. For invasion assays, the chambers were pre-coated with 50 μL of Matrigel (1:30 dilution in serum-free DMEM medium). Protocols were similar as described in the migration assays.

### A xenograft model of breast cancer

Fifteen athymic nude mice at the age of 6 weeks were randomly assigned as control group (n=5), 20 mg/kg cantharidin-treated group (n=5) and 40 mg/kg cantharidin-treated group (n=5). A xenograft model of breast cancer was established by injecting MDA-MB-231 cells (2×10^6^) into the right flank of each mouse. Mice from the experimental groups were injected with cantharidin (20 or 40 mg/kg) daily for 3 weeks. For each group of mice, tumor diameters (length, L and width, W) were monitored once a week. Tumor volume (TV) was calculated as TV=L×W^2^/2. Seven weeks after inoculation, all mice were sacrificed and tumors were dissected. The dissected tumors were weighed and photographed. All efforts were made to minimize suffering. Protocols for animal experiments were approved by the Ethics Committee from the First Affiliated Hospital of Zhejiang Chinese Medical University.

### Statistical analysis

Results are reported as means±SD. Student’s *t*-test was used to compare individual data with control values. Differences with a probability of P<0.05 were considered to be significant from control data.

## Results

### Cantharidin inhibited MDA-MB-231 cell growth in a dose- and time-dependent manner

**Figure 1.**
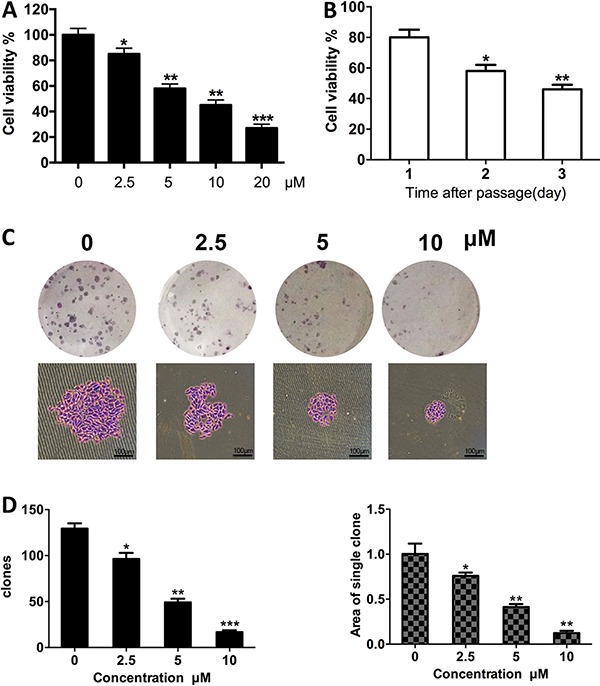
Cantharidin inhibited MDA-MB-231 cell growth in a dose- and time-dependent manner. *A*, MDA-MB-231 cells were assayed for cell viability determination with 2.5-20 μM cantharidin. Cells without cantharidin treatment were used as control. All groups of cells were cultured for 48 h. *B*, At a fixed dose (5 μM), cantharidin inhibited MDA-MB-231 cell viability in a time-dependent manner. *C*, Colony formation assays were performed using MDA-MB-231 cells treated with indicated doses of cantharidin. The clones were stained with crystal violet. *D*, In the colony formation assay, formed clones were counted manually for each group of cells (left panel). The size of each clone was also calculated and averaged (right panel). Data are reported as means±SD. *P<0.05; **P 0.01; ***P<0.001, compared to control (*t*-test).

Initially, the effects of cantharidin on MDA-MB-231 cell viability were assessed. Cantharidin concentrations were increased from 2.5 to 20 μM with a group of cells, and cells without any cantharidin treatment were used as control. After 48 h treatment, MDA-MB-231 cells were progressively less viable. The 50% lethal dose (LD50) was approximately 5 μM (Figure 1A). Based on this observation, MDA-MB-231 cell proliferation rates were then determined with a fixed dose of cantharidin treatment (5 μM). With the extended treatment time, cell viability decreased and was only half on day 3 than that on day 1 (Figure 1B). Furthermore, 0, 2.5, 5, and 10 μM doses of cantharidin were adopted in this study, and colony formation assays were performed. Colonies were smaller in size with the increasing cantharidin dose (Figure 1C). Quantitative analysis further revealed that colony numbers also decreased by increasing the doses of cantharidin (Figure 1D, left panel). Consistently, the average area of a single clone also decreased with the increase of cantharidin dose (Figure 1D, right panel). These data suggested that cantharidin inhibited MDA-MB-231 cell growth in a dose- and time-dependent manner.

### Cantharidin arrested cell cycle in G2/M phase in MDA-MB-231 cells

**Figure 2.**
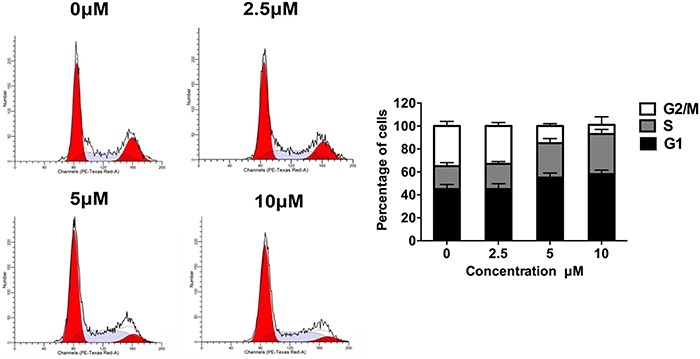
Cantharidin decreased the cell percentage in G2/M phase but increased that in S and G1 phases. This observation was also seen in higher doses of cantharidin treatment. Data are reported as means±SD.

Cell cycle was arrested in MDA-MB-231 cells with cantharidin treatment. More importantly, with the increasing doses of cantharidin, cell percentage in G2/M phase gradually dropped. In contrast, cell percentages in the S and G1 phases increased in cantharidin-treated MDA-MB-231 cells (Figure 2). These data suggested that cantharidin arrests cell cycle progression at G2/M phase in MDA-MB-231 cells.

### Cantharidin inhibited tumor growth in a xenograft model of breast cancer

**Figure 3.**
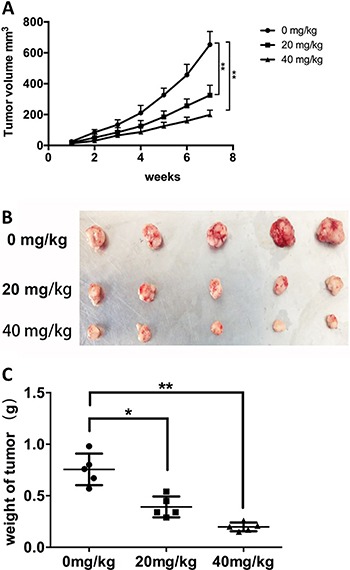
Cantharidin inhibited tumor growth in a xenograft model of breast cancer. *A*, Tumor volumes of xenograft model of breast cancer using MDA-MB-231 cells. The mice from each group were injected with cantharidin (20 or 40 mg/kg) daily for 3 weeks. The mice without cantharidin injection were used as control. *B*, Dissected tumors from each group of mice 7 weeks after inoculation. *C*, Weight of dissected tumors. Data are reported as means±SD. *P<0.05; **P<0.01 (*t*-test).

The mice without cantharidin injection were used as control. Tumor volumes in the three groups were different 4 weeks after inoculation. Control mice exhibited the largest tumor volumes 7 weeks after inoculation, whereas mice with cantharidin treatment exhibited significantly smaller tumor size (Figure 3A). After dissection, tumors from all groups were visually distinct in size. Tumors were the largest in the control mice and smaller in the cantharidin-injected mice (Figure 3B). The average weights of dissected tumors were also significantly different among the three groups. The mice injected with 20 mg/kg cantharidin bore tumors only 46.7% of the average weight of tumors in control mice, and 40 mg/kg cantharidin treatment remarkably shrunk the tumors even more compared to the control mice (Figure 3C). These data suggested that cantharidin could inhibit breast cancer growth *in vivo*.

### Cantharidin inhibited cell migration and invasion in MDA-MB-231 cells

**Figure 4.**
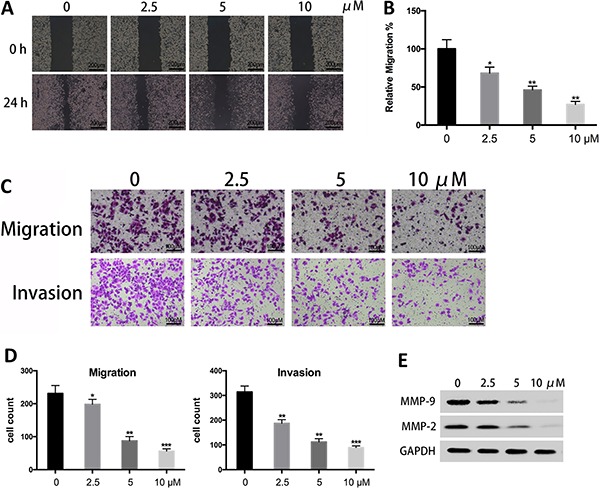
Cantharidin inhibited cell migration and invasion in MDA-MB-231 cells. *A*, Wound healing assay was performed to observe the effects of cantharidin treatment on MDA-MB-231 cell wound recovery. *B*, Quantification of the healed wound area showed that when MDA-MB-231 cells were treated with the LD50 dose (5 μM), wound closure was inhibited by approximately 50% compared to cantharidin-free MDA-MB-231 cells. This inhibition effect was even stronger when cells were treated with cantharidin at a dose of 10 μM. *C*, Representative images of transwell migration and invasion of cantharidin-treated MDA-MB-231 cells. *D*, Cell counts of transmigrated cells from the five photographed fields in each group. *E*, Western blot analysis of major MMP-2 and MMP-9 expressions in MDA-MB-231 cells with distinct doses of cantharidin. Data are reported as means±SD. *P<0.05; **P<0.01; ***P<0.001 compared to control (t-test).

Subsequently, MDA-MB-231 cells with distinct doses of cantharidin were subjected to migration and invasion assays. In the wound healing assay, higher doses of cantharidin inhibited the wound closure process (Figure 4A). The calculation of recovered wound area showed that when MDA-MB-231 cells were treated with the LD50 dose (5 μM), wound closure was inhibited by approximately 50% compared to cantharidin-free MDA-MB-231 cells. This inhibition effect was even stronger when cells were treated with cantharidin at a dose of 10 μM (Figure 4B). Consistently, transwell assays confirmed the above observations (Figure 4C). In the transwell migration assay, almost 240 control cells migrated to the lower surface, whereas 200 cells did only after 2.5 μM of cantharidin treatment; only 52 cells successfully migrated when cantharidin dose increased to 10 μM (Figure 4D, left panel). Similarly, the numbers of invaded cells in the transwell invasion assay were 182, 126, and 89, respectively, corresponding to 2.5, 5, and 10 μM cantharidin treatment doses. These data contrasted to the 308 successfully invaded control cells (Figure 4D, right panel). In accordance to the cantharidin-induced metastasis inhibition, the protein levels of major MMP-2 and MMP-9 were dose-dependently decreased by the cantharidin treatment (Figure 4E). The data of this study suggested a metastasis inhibition property of cantharidin in MDA-MB-231 cells.

### Cantharidin promoted the phosphorylated levels of MAPK signaling pathway

**Figure 5.**
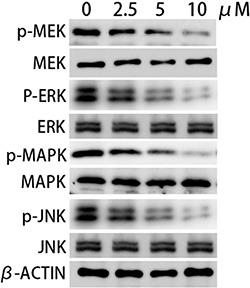
Total and phosphorylated levels of MAPK family members in MDA-MB-231 cells with distinct doses of cantharidin treatment, by western blot. Cantharidin promoted the phosphorylated levels of MAPK signaling pathway. ß-actin was developed as a loading control.

The protein levels of MAPK family members were detected next in MDA-MB-231 cells with distinct doses of cantharidin treatment. The total protein levels of MEK, ERK, MAPK, and JNK remained unchanged as cantharidin doses increased. However, the phosphorylated levels of MEK, ERK, MAPK, and JNK significantly decreased. MAPK signaling pathway is activated through phosphorylation by upstream kinases; thus, the decrease of the phosphorylation levels of MEK, ERK, MAPK, and JNK suggested that the MAPK signaling pathway was inactivated by cantharidin treatment (Figure 5).

## Discussion

Based on previous findings of cantharidin as the active constituent of the Chinese medicine *Mylabris* (8,10–12,14,15,17,20), the possible therapeutic values of cantharidin against breast cancer and underlying pathways associated with this anticancer activity were investigated in this study.

Our data showed that cantharidin inhibited MDA-MB-231 cell viability and clonogenic potential in a dose-dependent manner. Cantharidin significantly inhibited tumor volume and weight in the xenograft model of breast cancer *in vivo*. Furthermore, cell cycle analysis revealed that cells were increasingly accumulated in the G2/M phase with the increased cantharidin dose. The cell cycle arrest at the G2/M phase reinforced the tumor growth inhibition effect by cantharidin. Hence, these *in vitro* and *in vivo* data strongly suggested that cantharidin had potent anticancer growth activity in breast cancer. In addition to cell growth inhibition, cantharidin inhibited cell migration and invasion in MDA-MB-231 cells. Importantly, MMP-2 and MMP-9 are zinc-dependent proteinases that are involved in the degradation and remodeling of extracellular matrix. MMP-2 and MMP-9 can degrade matrix collagen and basement membrane (22,23) and correlate with the invasive and metastatic properties of cancer (24,25). The decreases of MMP-2 and MMP-9 expression by cantharidin further supported the cantharidin-mediated inhibition of cell migration and invasion in MDA-MB-231 cells. Therefore, cantharidin potently inhibits cell growth and metastasis of breast cancer cells. The anticancer activity of cantharidin may suggest the potential of using cantharidin as a therapeutic reagent against breast cancer.

Since cantharidin is a potent and selective inhibitor of PP2A (8), it is supposed to be functionally associated with phosphorylation activity. Indeed, the data of this study showed that the phosphorylation levels of MAPK signaling (p-ERK and p-JNK, and p-p38 MAPK) were decreased by cantharidin treatment in MDA-MB-231 cells, confirming the hypothesis that cantharidin might regulate the MAPK signaling pathway in breast cancer. The mammalian MAPK family consists of ERK, p38, and JNK with each member having several isoforms: ERK1 to ERK8; p38α (MAPK14), p38ß (MAPK11), p38γ (MAPK12), and p38δ (MAPK13); and JNK1 (MAPK8), JNK2 (MAPK9), and JNK3 (MAPK10). Each MAPK signaling cascade consists of at least three layers: an MAPK kinase kinase (also known as RAF), an MAPK kinase (also called MEK), and a MAPK (26). Activated MAPKs phosphorylate numerous substrates and thereby regulate many important cellular processes, such as differentiation, proliferation, survival, and cell adhesion (27). Recent data indicate that p38 MAPK-driven MAPKAPK2 regulates the invasion of bladder cancer by modulating MMP-2 and MMP-9 activities (28). In this study, cantharidin dose-dependently decreased the phosphorylation levels of ERK, JNK, and p38 MAPK, as well as MEK, suggesting that cantharidin regulated the MAPK signaling pathway. Consistent with the results of this study, another study has shown that cantharidin inhibits cell migration and invasion of human lung cancer NCI-H460 cells via UPA and MAPK signaling pathways (29). However, a previous report has found that only p38 and JNK/MAPK are involved in cantharidin-mediated cell apoptosis in leukemia U937 cells (28). ERK was intriguingly not associated with the above activity, which contradicted with the data of this study. This controversy might be due to the different cancer types; and if this is true, distinct mechanisms might underlie cantharidin-mediated anticancer activity in different cancer types. However, further research needs to be done.

Overall, cantharidin exerted a potent inhibition of growth and metastasis in breast cancer. The anticancer activity of cantharidin may be associated with MAPK signaling pathway. Our data highlight the possibility of using PP2A as a therapeutic target for breast cancer treatment.
